# Production of Sterilizing Agents from *Calendula officinalis* Extracts Optimized by Response Surface Methodology

**DOI:** 10.1155/2015/789732

**Published:** 2015-05-07

**Authors:** Fatih Mehmet Goktas, Bilgesu Sahin, Sibel Yigitarslan

**Affiliations:** Chemical Engineering Department, Engineering Faculty, Suleyman Demirel University, West Campus, 32000 Isparta, Turkey

## Abstract

The aim of this study was to produce hand sterilizing liquid and wet wipes with the extracts of *Calendula officinalis*. Since this plant has well known antimicrobial activity due to its phytochemical constituents, the increase in the extraction yield was chosen as the principle part of the production process. To achieve the maximum yield, parameters of solid-to-liquid ratio, extraction temperature, and time were studied. The optimum conditions were determined by response surface methodology as 41°C, 7 h, and 3.3 g/200 mL for temperature, time, and solid-to-liquid ratio, respectively. The yield achieved at those conditions was found to be 90 percent. The highest amounts of flavonoids were detected at optimum, whereas the highest triterpene and saponin constituents were determined at different design points. The microbial efficiencies of extracts were determined by the inhibition of the growth of selected microorganisms. Different dilution rates and interaction times were used as parameters of inhibition. Not any of the constituent but symbiotic relation in-between reached the highest inhibition of 90 percent. The pH values of the extracts were 5.1 to 5.4. As a result, the extraction of *Calendula officinalis* at the optimum conditions can be used effectively in the production of wet wipes and hand sterilizing liquid.

## 1. Introduction

Herbal plants are popular remedies for diseases used by most of the world's population. Phytochemicals exert positive effects on human health and thus they are important compounds found in medicinal plants, such as* Calendula officinalis, Pandanus amaryllifolius* [1],* Taraxacum officinale* [2], and* Epimedium brevicornum* [3]. The type and the amount of the phytochemicals depend on the medicinal plant.* Calendula officinalis* is a herb with yellow-orange flowers, mostly seen in the Mediterranean region [1]. It has a long history for safe use as food and as a medicine [2–4]. Its usage for the production of drug comes from its phytochemical constituents, such as alkaloids, carbohydrates, fats and oils, saponins, triterpenes, flavonoids, phenols, tannins, and diterpenes [5, 6]. 7.71 g of total carotenoids, 4.33 g of total amino acids, 13.52 g of total carbohydrates, and 17.20 g of total fat and oil were detected per 100 g of this plant on dry basis [6]. Due to the approval of GRAS (generally recognized as safe) statue by Food and Drug Administration, it reached a high economic value and is widely used in cosmetics as well as other application areas [1].

It is well documented that* Calendula officinalis* L. has an antimicrobial activity. In the twenty-first century investigations, results showed that it was effective against all 23 clinical fungi strains tested [1], against 18 strains of anaerobic or facultative aerobic periodontal bacteria [7], and also against 19 clinical bacterial pathogens [8]. In those researches, the plant extracts were prepared by different types of solvents as water [1, 9], hexane [1], methanol [7, 8], ethanol [8], and pet ether [9]. Since the extraction temperatures, times, and solid-to-solvent ratios were also different, the yield of extraction was found in the range of 17.4 to 25.8 g/100 g [1, 8, 9]. When compared, the most efficient solvent in the context of antibacterial effect and the yield of extraction was determined as water [9].

Extraction is the most important unit operation since it strongly affects the type and the amounts of phytochemical constituents. The most frequently used method for extraction of those compounds is conventional extraction achieved by Soxhlet apparatus. However, the requirement of prolonged extraction at high temperature results in the degradation of some phytochemicals and high costs of energy [1]. Thus, any effort for optimizing the most appropriate extraction condition is valuable to achieve the highest yield. Response surface method is a collection of statistical and mathematical techniques that is used to analyze several independent variables and also interactive effects among the variables on the response. This method has been used in several optimizations, that is, adsorption [10], extraction [1–3, 11, 12], and production processes [13–16]. The advantages of the method are reduced number of experimental runs, cost, and time compared to other methods [14–16].

In the literature survey, there was not any study found about the optimization of the extraction of phytochemicals from* Calendula officinalis* by the novel method of optimization, namely, response surface methodology.

Since the increase in the yield of extraction increases the phytochemical constituent and thus the antibacterial and antifungal properties, the objective of the present research was to optimize the extraction conditions of phytochemical constituents of* Calendula officinalis* L. and to examine the usability of the extracts in the production of wet wipes and hand sterilizers. The effects of variables including extraction time, temperature, and solid-to-liquid ratio on the extraction yield were investigated by three-level-three-parameter Box-Behnken design (BBD). The optimum conditions were determined by a computer program using response surface methodology. The components of the extracts were determined by respective methods of the constituent. The extracts obtained at the optimum conditions were used in the production of wet wipes and hand sterilizers. Finally, the effectiveness of the products was analyzed.

## 2. Materials and Methods

*Calendula officinalis* L. and sodium alginate were purchased from a commercial herb seller and Kimbiotek Inc., respectively.

In the preliminary investigations, the potential design factors of extraction, such as solvent type, temperature, mixing rate, and amount of solvent and solid, were determined by using the information obtained at the end of detailed investigations of the corresponding literature. Then, held-constant factors, allowed-to-vary factors, and the response variable were determined considering the aim of the research. For example, the solvent type was determined as water (held-constant factor) since the aim was to produce sterilizing agents usable both for adults and for babies, whereas temperature and duration of extraction were allowed-to-vary factors. The response variable was selected as the yield of extraction due to the fact that all extractable phytochemicals will be at maximum value. After that, allowed-to-vary factors were investigated by designing simple comparative experiments. The most effective three parameters and their possible interactive effects were determined by comparing the extraction yields of those comparative experiments. Finally, the levels of the parameters were chosen as center point values of the optimization of extraction. In order to demonstrate their interactive effects clearly response surface optimization technique was applied.

Optimization studies were carried out by studying the effects of three variables including extraction temperature, time, and solid-to-liquid ratio. The chosen independent variables were coded according to

(1) $$\begin{array}{c} x_{i}=\frac{\left(x_{i}-x_{0}\right)}{\Delta x}, \end{array}$$

where *x*_*i*_ is the dimensionless coded value of *i*th independent variable, *x*_0_ is the value of *x*_*i*_ at the center point, and Δ*x* is the step change value. The behavior of the system is explained by the following second-order polynomial model:

(2) $$\begin{array}{c} Y=\beta_{0}+\overset{k}{\underset{i=1}{\sum }}\beta_{i}x_{i}+\overset{k}{\underset{i=1}{\sum }}\beta_{ii}x_{i}^{2}+\overset{k-1}{\underset{i=1}{\sum }}\;\overset{k}{\underset{j=2}{\sum }}\beta_{ij}x_{i}x_{j}+\varepsilon , \end{array}$$

where *Y* is the predicted response, *x*_*i*_, *x*_*j*_,…, *x*_*k*_ are input variables, which affect the response *Y*, *x*_*i*_^2^, *x*_*j*_^2^,…, *x*_*k*_^2^ are the square effects, *β*_0_ is the intercept term, *x*_*i*_ *x*_*j*_, *x*_*j*_ *x*_*k*_ and *x*_*i*_ *x*_*k*_ are the interaction effects, *β*_*i*_  (*i* = 1,2,…, *k*) is the linear effect, *β*_*ii*_  (*i* = 1,2,…, *k*) is the squared effect, *β*_*ij*_  (*i* = 1,2,…, *k*) is the interaction effect, and *ε* is the random error [14–16].

The Design-Expert 9.0 (Stat-Ease Inc., Minneapolis, MN, USA) software was used for regression and graphical analysis of the experimental data to fit the equations developed and evaluation of their statistical significance. BBD is frequently used under response surface method due to its suitability to fit quadratic surface that usually works well for process optimization. Design of 15 experiments consisting of three replicates at the central point was employed to the second-order and also cubic polynomial model. The optimum values of the selected variables were obtained by solving the regression equation at desired values of the process responses as the optimization criteria. Each of the parameters was coded at three levels: −1, 0, and +1. The range and level of the variables in coded units for response surface methodologies were given in Table 1.

Extraction was realized batch-wise in a 250 mL Erlenmeyer flask fitted in an isothermal shaker (Memmert). After making sure that the temperature of the solvent had reached the desired value (*x*_1_), determined amount of dry plant was added (*x*_2_). At that time extraction was started and at the end of the specified extraction time (*x*_3_) and the content of the flask was filtered through 110 mm filters (FilterLab). Filtered samples were used for further analysis.

The yield of extraction was determined by dry matter analysis. 1 mL of each sample was dried in an oven at 110°C during 12 h. The yields were calculated by inserting the mass differences before and after the drying in (3). The value of total dry matter content of the plant (42.76 g) was taken from the literature [6]:

(3) $$\begin{array}{c} \%\text{Yield}=\frac{\text{dry matter extracted}\;\text{(g)}}{42.76\;\left(\text{g}\right)}\times 100. \end{array}$$

Characterization of the extracts was investigated considering their triterpene, total flavonoid, and saponin contents. The general procedure of spectrophotometric analysis of triterpenes and total flavonoids was applied [9]. Saponins constituents of the extracts were detected according to the foam test [9].

Since the appearance of golden yellow color indicates the presence of triterpenes [9], the colors of the extracted samples were determined by spectrophotometer (LANGF, DR 5000) at 400 nm. Those absorbance values were used to compare the triterpene amounts present in the corresponding extracts.

Total flavonoid content of the extracts was estimated by aluminium chloride colorimetric method. After the extraction, 1 mL of extracts was diluted with 4 mL of distilled water. 5%, 0.3 mL NaNO_2_ solution was added to each sample. After 5 min %10, 0.4 mL of AlCl_3_ and then at 6 min 1.0 M 2 mL NaOH solution were added. Absorbances of the solutions were read at 475 nm.

The wet wipes were prepared by allowing dry towels to absorb 6 mL of diluted extraction samples. The hand sterilizing liquid was prepared by the addition of 9 g sodium alginate to 600 mL of diluted extraction samples.

In order to determine the antimicrobial activity of the extracts different dilution ratios (10 and 30 mL/L of solution) were added to the usual growth medium of mixed microorganism cultures. In the culture, there were 6 subspecies of* Bacillus* and 3 subspecies of* Lactobacillus* types present. The growth in those of extracts was analyzed by measuring their absorbance at 420 nm during 30 h.

## 3. Results and Discussion

### 3.1. Regression Model and Optimization

The coded variables and their respective yields were entered into the software (Table 2). The suggested models were produced by using Box-Behnken design. Applying all the statistical tests, the reduced cubic model was found to be the best model that describes the surface of yield. The statistical significance of the model was evaluated by analysis of variance (ANOVA) as presented in Table 3. Since the larger the magnitude of the *F*-value and the smaller the *p* values, the more significant the corresponding coefficient, the most effective parameter on the extraction yield was found to be solid-to-liquid ratio. Extraction temperature was found more effective than extraction time.

**Figure 1:**
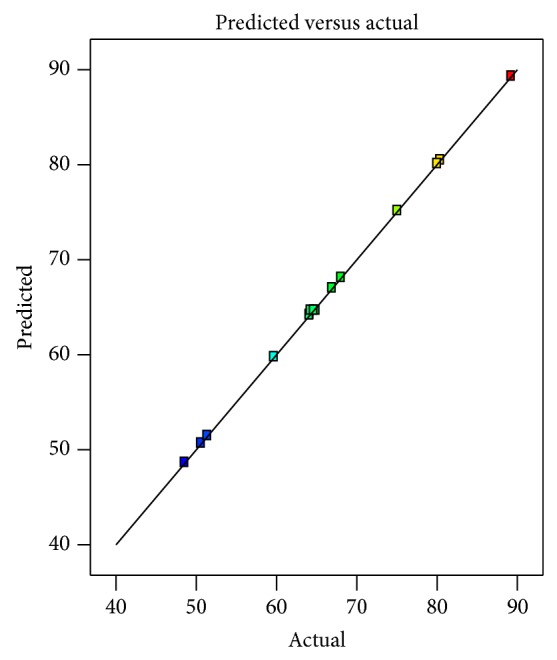
Comparison of predicted and actual values of the study.

In order to check the model found, the predicted and actual yield values were compared. As can be seen from Figure 1, good agreement was observed. The regression coefficients were also in agreement with this conclusion (Table 3). Thus, the empirical relation explaining the surface was found as in

**Figure 2:**
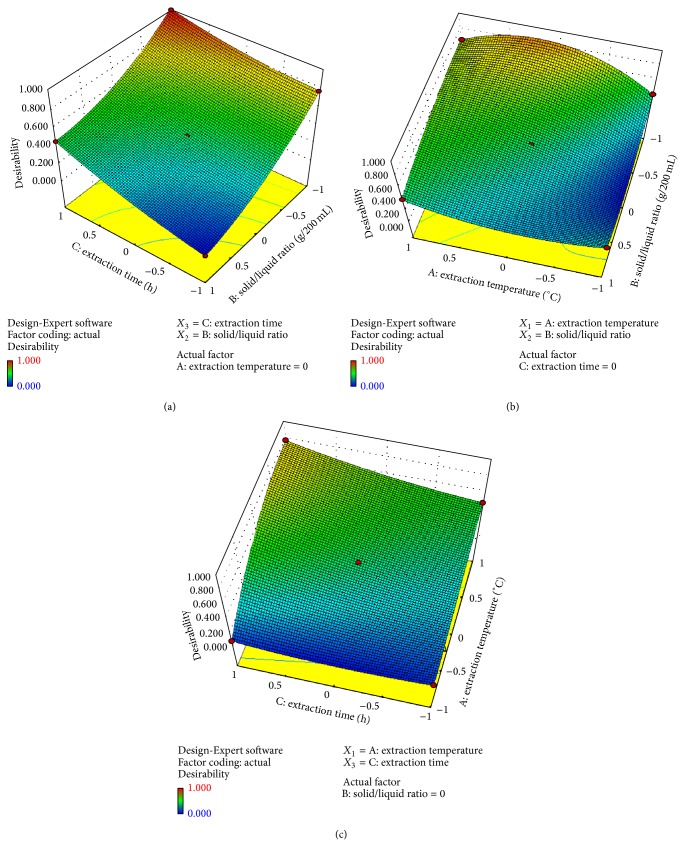
Three-dimensional response surfaces of the effective parameters on the extraction process.

(4) Yield=64.65+11.61x1−13.44x2+6.9x3−1.63x1x2+2.89x1x3+2.28x2x3−3.71x12+4.87x22+1.73x32+9.58x12x2−3.61x12x3−7.78x1x22,

where *x*_1_, *x*_2_, and *x*_3_ were the coded values of the parameters defined in Table 2. Response surfaces were obtained by the respective button of the software. The parameters and their interactive effects were shown in Figure 2. The increase in extraction yield was observed with decreasing the solid-to-liquid ratio (a), while increasing the temperature up to a specific value less than 50°C (b) and the extraction time as soon as possible (c). The requirement of high temperature and long time may be due to different types and amounts of antimicrobial compounds present in plant to be extracted into the solvent. In order to determine the values of the parameters that accommodate the optimum conditions, numerical optimization section and desirability function were used. All of the variables were chosen “in range” statue except the yield chosen as “max.” The values of those parameters were evaluated by a computer and found as follows:

 Extraction temperature: 41.25°C;
 Extraction time: 7 h;
 Solid-to-liquid ratio: 3.3 g/200 mL.

### 3.2. Characterization of Extracts

At the end of the optimization, the extraction was done at the determined optimum values of the parameters and the yield of extraction was calculated. The 90% yield of extraction calculated by the software was approved by the experiments duplicated. The differences between those yields were not found to be statistically significant.

The triterpene, flavonoid, and saponin components of the best three extracts were analyzed and compared. It was observed that all of the extracts had all of the components examined. The highest saponin content was determined in the extracts obtained under the following conditions: 37.5°C, 3.3 g/200 mL solid-to-liquid ratio, and 1 h of extraction. When flavonoid components were compared, it was seen that the highest amounts were found in the extracts produced at optimum conditions; 41°C, 3.3 g/200 mL solid-to-liquid ratio, and 7 h of extraction. Triterpenes were found dominant in the extracts obtained at 37.5°C temperature and 16.5 g/200 mL solid-to-liquid ratio during 7 h extraction. Finally, the pH values of the extracts were in the range of 5.1 to 5.4.

### 3.3. Products of Extraction

**Figure 3:**
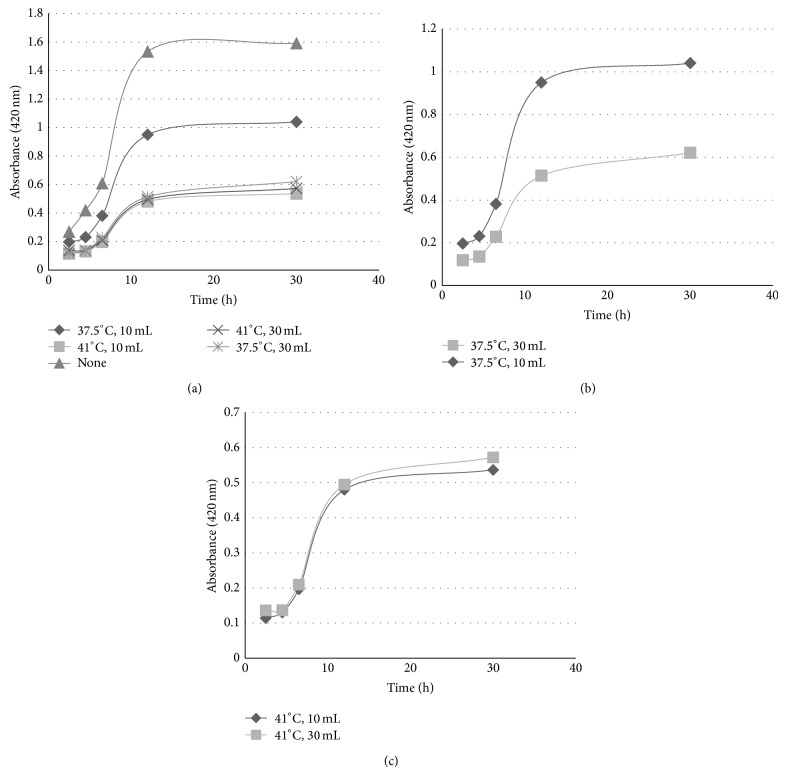
Microbial growth in different extracts added to liquid mediums.

In the production of wet wipes and hand sterilizer, different dilution rates (10 and 30 mL/L of solution) and the best two different extracts were used. The growth of* Bacillus* subspecies in the presence of diluted extract containing liquid medium was investigated during 30 hours (Figure 3(a)). It was observed that the inhibition of microbial growth was strongly dependent on the concentration of the extracts obtained at 37.5°C (Figure 3(b)). Nearly half of the microorganism growth can be easily inhibited by those extracts. However, the extracts obtained under optimum conditions inhibited the microorganism growth independent of its concentration (Figure 3(c)). In the medium containing the extracts obtained under optimum conditions, there was no significant microbial increase during 5 hours. The best dilution rate was determined as 10 mL of extract at optimum conditions/L of solution for the inhibition of the growth of the pathogens by 90%.

## 4. Conclusion

In this study, in order to develop an equation describing the relation between the extraction yield and three variables shown in Table 1, a Box-Behnken Design was conducted. Reduced cubic model was used to correlate the variables to the response. The model was found significant and the empirical equation was used for construction of response surfaces.

Extraction of phytochemicals was found very sensitive to solid-to-liquid ratio and temperature. 90% yield of extraction was achieved at optimum conditions determined by numerical optimization and confirmed by the experiments. This value which was equal to 38.4 g/100 g of dry herb was the highest when compared to the given values of previous studies [1, 8, 9]. The components compromising the phytochemicals were the same as they were declared in the literature. Since both of the products (wet wipes and hand sterilizer) produced by the dilution of extracts could inhibit %90 of the growth of microorganisms investigated, it was concluded that the extracts of* Calendula officinalis* at the optimum conditions determined can be used in the production of wet wipes and hand sterilizers, effectively.

## Figures and Tables

**Table 1:** Experimental ranges and levels of the independent variables.

Variables	Range and level		
	−1	0	+1
Extraction temperature, °C (*x*_1_)	25	37.5	50
Solid/liquid ratio, g/200 mL (*x*_2_)	3.3	10.0	16.5
Extraction time, h (*x*_3_)	1	4	7

**Table 2:** The coded variables and experimental yield values in the study.

Run	*x*_1_ (°C)	*x*_2_ (g/200 mL)	*x*_3_ (h)	Yield (%)
1	−1	1	0	59.75
2	−1	0	1	51.45
3	0	0	0	64.94
4	0	1	1	66.98
5	1	0	1	80.45
6	0	−1	1	89.29
7	0	1	−1	48.62
8	−1	−1	0	64.20
9	1	1	0	64.15
10	0	0	0	64.31
11	1	−1	0	75.12
12	−1	0	−1	50.65
13	1	0	−1	68.10
14	0	−1	−1	80.07
15	0	0	0	64.69

**Table 3:** Analysis of variance (ANOVA) for the reduced cubic model.

Source	Sum of squares	df	Mean square	*F* value	*p*-value Prob > F
Model	1837.83	12	153.15	1521.89	0.0007

*x*_1_	539.40	1	539.40	5360.06	0.0002
*x*_2_	722.53	1	722.53	7179.87	0.0001
*x*_3_	190.16	1	190.16	1889.67	0.0005
*x*_1_ *x*_2_	10.63	1	10.63	105.61	0.0093
*x*_1_ *x*_3_	33.35	1	33.35	331.41	0.0030
*x*_2_ *x*_3_	20.88	1	20.88	207.53	0.0048
*x*_1_^2^	50.81	1	50.81	504.90	0.0020
*x*_2_^2^	87.50	1	87.50	869.45	0.0011
*x*_3_^2^	10.99	1	10.99	109.23	0.0090
*x*_1_^2^ *x*_2_	183.74	1	183.74	1825.88	0.0005
*x*_1_^2^ *x*_3_	26.03	1	26.03	258.64	0.0038
*x*_1_ *x*_2_^2^	121.13	1	121.13	1203.72	0.0008

Pure error	0.20	2	0.10		

Cor total	1838.03	14			

^∗^ *R*-squared = 0.9999; adjusted *R*-squared = 0.9992; Std. Dev. = 0.32.
